# Nature and Consequences of Biological Reductionism for the Immunological Study of Infectious Diseases

**DOI:** 10.3389/fimmu.2017.00612

**Published:** 2017-05-31

**Authors:** Ariel L. Rivas, Gabriel Leitner, Mark D. Jankowski, Almira L. Hoogesteijn, Michelle J. Iandiorio, Stylianos Chatzipanagiotou, Anastasios Ioannidis, Shlomo E. Blum, Renata Piccinini, Athos Antoniades, Jane C. Fazio, Yiorgos Apidianakis, Jeanne M. Fair, Marc H. V. Van Regenmortel

**Affiliations:** ^1^Center for Global Health, Division of Infectious Diseases, School of Medicine, University of New Mexico, Albuquerque, NM, United States; ^2^National Mastitis Center, Kimron Veterinary Institute, Bet Dagan, Israel; ^3^Environmental Assessment, U.S. Environmental Protection Agency, Seattle, WA, United States; ^4^Department of Fisheries and Wildlife, Michigan State University, East Lansing, MI, United States; ^5^Human Ecology, Centro de Investigación y de Estudios Avanzados (CINVESTAV), Mérida, México; ^6^Department of Internal Medicine, School of Medicine, University of New Mexico, Albuquerque, NM, United States; ^7^Department of Biopathology and Clinical Microbiology, Aeginition Hospital, Medical School, National and Kapodistrian University of Athens, Athens, Greece; ^8^Department of Nursing, Faculty of Human Movement and Quality of Life Sciences, University of Peloponnese, Sparta, Greece; ^9^Department of Veterinary Medicine, University of Milan, Milan, Italy; ^10^Department of Computer Science, University of Cyprus, Nicosia, Cyprus; ^11^Department of Biological Sciences, University of Cyprus, Nicosia, Cyprus; ^12^Los Alamos National Laboratory, Biosecurity and Public Health, Los Alamos, NM, United States; ^13^School of Biotechnology, Centre National de la Recherche Scientifique (CNRS), University of Strasbourg, Strasbourg, France

**Keywords:** methods, host–microbe interactions, reductionism, non-reductionism, pattern recognition

## Abstract

Evolution has conserved “economic” systems that perform many functions, faster or better, with less. For example, three to five leukocyte types protect from thousands of pathogens. To achieve so much with so little, biological systems combine their limited elements, creating complex structures. Yet, the prevalent research paradigm is reductionist. Focusing on infectious diseases, reductionist and non-reductionist views are here described. The literature indicates that reductionism is associated with information loss and errors, while non-reductionist operations can extract more information from the same data. When designed to capture one-to-many/many-to-one interactions—including the use of arrows that connect pairs of consecutive observations—non-reductionist (spatial–temporal) constructs eliminate data variability from all dimensions, except along one line, while arrows describe the directionality of temporal changes that occur along the line. To validate the patterns detected by non-reductionist operations, reductionist procedures are needed. Integrated (non-reductionist and reductionist) methods can (i) distinguish data subsets that differ immunologically and statistically; (ii) differentiate false-negative from -positive errors; (iii) discriminate disease stages; (iv) capture *in vivo*, multilevel interactions that consider the patient, the microbe, and antibiotic-mediated responses; and (v) assess dynamics. Integrated methods provide repeatable and biologically interpretable information.

## Reductionism and Its Historical Background

Numerous calls have asked for new methods applicable to infectious disease research. They are motivated by: (i) insufficient information on host-microbial interactions; (ii) obsolete microbial classifications—including “pathogenic” and “non-pathogenic” species; (iii) the need to distinguish “infectiveness” from “virulence”; (iv) the apparent end of the antibiotic era; and (v) requests for more reliable medical diagnoses than those based on research involving a single factor (1–12). Hoping to foster biologically grounded methods, this mini review describes the properties of infectious disease-related data, as well as *reductionism*—the belief that biology can be reduced to few and simple variables.

Three types of reductionism (ontological, epistemological, and methodological) have been described (13). While the first two types involve *abstract* (non-measurable) concepts, reductionist methods utilize *concrete* (measurable) operations. Because conceptualizations precede operationalizations, invalid concepts may promote invalid methods. Therefore, the validity of methods already applied or expected to be used in infectious diseases, in the future, should be determined.

Biology has adopted methods used in Physics (14, 15). Most notably, reductionist approaches have been followed in the field of molecular biology (12, 15–17). While such a fact should not be construed to imply that physics is reductionist *per se*, the opposite can be emphasized: in contrast to many physical systems, biological systems are generally complex, requiring approaches that far exceed the study of isolated component parts (18).

While biological *reductionism* has been successful, it has also been associated with failure and cognitive stagnation (12). For instance, after 20,000 publications on sepsis, only one new drug has been legally approved (1, 19). At least two facts suggest that reductionism has hampered vaccine development: (i) more than a thousand synthetic peptide vaccines have been generated but none has been approved, and (ii) reverse vaccinology has not yet produced effective HIV vaccines (10, 13, 17). The high percentage (up to 42%) of research funding reported to be wasted may be due to inadequate methods, which include reductionism (15, 20).

Reductionism has prevailed since Descartes published “The discourse on the method” (15). It is based on *deductions*, as when Halley *predicted*, in 1705, that a comet would be seen in 1758 (21). In contrast, Biology thrives on *inductions* made *after* data are collected (17).

While Descartes has been viewed as the founder of reductionism (Movie S1 in Supplementary Material), that is not what he proposed: in 1637, he described *four rules*, reductionism being only the second rule of a method that also included (i) data analysis (first rule), (ii) integration (the third rule), and (iii) comprehensive assessments (the fourth rule). Descartes’ third and fourth rules have not yet been applied in Biology (22).

Two centuries later, Claude Bernard championed biomedically grounded methods (23). He proposed to study the *internal milieu*—today known as *homeostasis* or feedback processes. Later, von Bertalanffy showed that biological systems are not closed, but open (24). Thus, “internal” and “external” factors—e.g., host–microbial interactions—should be investigated.

## Reductionism-Related Errors and Information Loss

The difference between *immunogenicity* and *antigenicity* illustrates why reductionism, in Biology, is failure prone (17). *Antigenicity* is simply the chemical capacity of a protein (e.g., a viral protein) to bind some preexisting antibodies. In contrast, *immunogenicity* is the *in vivo* capacity of the immune system to respond against an immunogen (e.g., a viral antigen) when it is introduced into an animal with the purpose of producing antibodies directed against the antigen. While the *complex* immune system elicits poly-reactive antibodies that recognize numerous antigens, only some antibodies may neutralize the infectivity of the pathogen (25).

The previous concepts explain why reductionist attempts to design vaccine immunogens by molecular engineering usually fail (17). Two errors explain such failures: (i) because the neutralization capacity of a polyclonal antiserum depends on many and different antibodies, *outcomes cannot be predicted from the structure of any one antibody*; and (ii) because *in vivo* interactions involve the pathogen, antibodies, and some but not all host cells, *outcomes depend on multifactor, in vivo relationships*, which are not considered by synthetic approaches (26–31).

Reductionism is unintentionally practiced in many fields. For example, computer sciences are influenced by the “*curse of dimensionality*”—a term that refers to the large number of calculations that computers may need to perform (32). To avoid millions of calculations, the number of *dimensions* to be analyzed may be *reduced* (33). Fields that *reduce dimensions* lose valuable information, e.g., in epidemiology, controlled trials do not assess comorbidities, even though they play major roles in infectious diseases (34–37).

Some quantitative traditions also limit the analysis of host–microbial interactions, e.g., *correlation analysis* neither explains nor predicts (38). *Network analysis* (a static method) cannot capture dynamics (39). While classic statistics assume *linearity, independence*, and also regard as *constant* the *meaning* of any numerical assessment, these beliefs do not apply to immunomicrobial data: leukocytes are neither linearly distributed nor independent, and numbers derived from immune cells may have different interpretations at different times. That is, leukocyte data can be non-informative or *ambiguous* (40, 41).

Errors also happen due to inadequate *procedures*—such as those commonly used with “*compositional*” data (e.g., leukocyte percentages). Because the same ratio value may be found in different biological conditions, simple leukocyte ratios induce ambiguity (42–44). Errors are also generated by *dichotomization*: when a cutoff divides continuous data (e.g., leukocyte percentages) into two subsets and discontinuous labels—e.g., “infection-negative” and “-positive”—are assigned to each subset, false-positive and -negative errors invariably occur (45).

## Toward Remedial Strategies (I): The Properties of Infectious Disease-Related Data

Infectious disease-related data reveal, at least, four properties: (i) *circularity*, (ii) *heterogeneous temporal scales*, (iii) *ambiguity*, and (iv) *hidden structures* (41, 43, 44). Understanding their features or consequences may prevent errors and information loss.

Data *circularity* is detected when three-dimensional (3D) interactions are explored—which become four-dimensional (4D) when time is also measured (43). The analysis of dynamics matters because *what has occurred in the past will*—or may—*be repeated in the future* (46). Because the circularity of temporal data shows neither beginning nor end, dynamics cannot be studied with approaches that utilize confidence intervals (43, 45, 47).

Because some processes occur within minutes or hours (e.g., early antimicrobial responses), while other responses—e.g., healing—take place over days or weeks (48, 49), the use of identical chronological units promotes *information loss*: any one unit may be too large or too small to detect all immune functions. To capture *heterogeneous temporal scales*, “biological” (not chronological) units may be needed. Two examples of “biological” units include: (i) the increased neutrophil values that characterize early inflammatory responses (expressed as higher neutrophil/lymphocyte [N/L] ratio values), and (ii) the augmented mononuclear cell/neutrophil [MC/N] values (typically observed in the resolution phase). Such well-conserved immune profiles could act as the biological equivalents of “early and late hours” (43).

*Ambiguity* results when the same numerical value of the same variable is found in different biological conditions (41). Also known as *spatial relativity*, it occurs when data collected over short time frames (e.g., 1 day before and 1 day after a new infection develops) occupy a large portion of the space under analysis, and *vice versa* (50).

Because, in 3D/4D space, the number of data combinations may approach infinity, some data structures may be “compressed”, i.e., unobservable (51, 52). Hence, *hidden information* is a common consequence of the combinatorial properties that characterize Biology.

## Toward Remedial Strategies (II): Methodological Foundations

Three traditions facilitate method development: (i) those grounded on *theory*, (ii) methods expressed with a mathematical language (“*modeling*”), and (iii) approaches that do not consider theories or models, but “*mechanistic”* (i.e., limited) explanations (14). Thus, methods that capture a major biological theory in their operations can be more explanatory than alternatives.

Accordingly, methods centered on “organizing principles” have been proposed (12, 53). New methods could capture critical (system-level) *biological properties*—not features derived from convenience or borrowed from other fields—e.g.: (i) “*one-to-many/many-to-one” combinatorial* features (39), (ii) *complexity* (15, 16), and (iii) *three-/four-dimensional dynamics* (41, 44). These properties are not necessarily different: they may express the same phenomena.

The “*one-to-many/many-to-one”* feature has two presentations: (i) any one element (e.g., a cell type) can participate in two or more functions, and (ii) to be performed, any one function requires two or more elements. For instance, macrophages promote or destroy neutrophils and, together with lymphocytes, conduct *complex* functions—for instance, antigen activation (54).

While *complexity* may be indefinable and defy human understanding (52, 55, 56), four features describe it: (i) *emergence*, (ii) *irreducibility*, (iii) *unpredictability*, and (iv) *autonomy*. *Autonomy* means non-linearity: effects are *not proportional* or *linear* (57). *Emergent* features—e.g., those of virulence—are observed when a highly complex structure is assembled (58). Emergence (distinct, non-random patterns) may be detected using dimensionless numbers derived from leukocyte data, which create complex (although hypothetical) data structures (52, 59). Because *emergence* can neither be *reduced* to, nor *predicted* from isolated variables, to detect it, “top-down” (not only “bottom-up”) approaches are needed (12, 13, 16, 58).

While reductionism measures *variables* in isolation, combinatorial (non-reductionist) approaches capture *spatial–temporal relationships*. Distinct patterns emerge when, in 3D/4D space, dimensionless indicators *converge*, not when a single variable changes (60, 61). Because multidimensional pattern recognition does not require numerical cut-offs, it prevents errors associated with *dichotomization*, “*compositional”* data, *circularity*, and *ambiguity* (39, 40, 44).

While “organizing properties” are necessary, they are not sufficient to prevent two problems: (i) *data variability* and (ii) the *multiple scale*s of *temporal data* (48). Both problems may be addressed with structures that reveal a *single* (one data point-wide) *line* of observations. Such structures eliminate variability from all dimensions—except along the line—and detect temporal changes that occur along the line, even when such changes are numerically small and/or the individuals being tested include “slow” and “fast” responders (41, 51).

## Visualization of Reductionist and Non-Reductionist Paradigms

**Figure 1:**
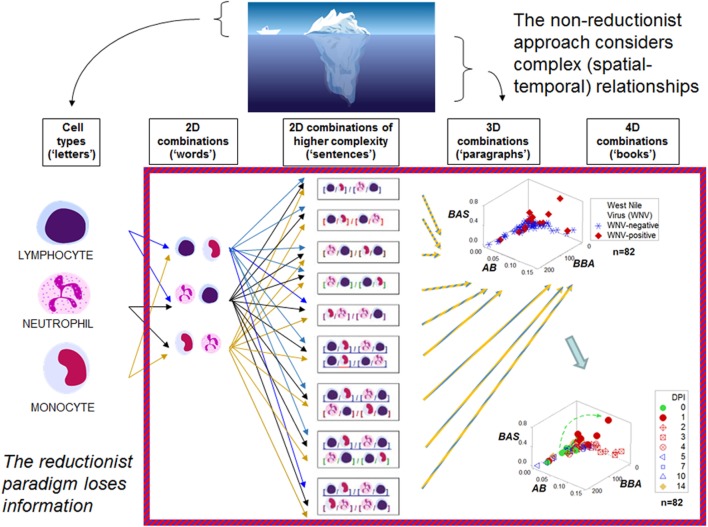
**Reductionist and non-reductionist views**. An iceberg is used to describe (i) reductionism (the “tip of the iceberg”, i.e., an easily measured entity that does not express all the available information), and (ii) non-reductionism (a combinatorial and spatial–temporal analysis of biological complexity and dynamics, i.e., the area “below the surface”). These concepts are illustrated with an analogy that refers to written language. While simple elements (“letters”) lack meaning, combinations of increasing complexity (“words”, “sentences”, “paragraphs”, “books”) exhibit distinct patterns that facilitate the partitioning of the data into subsets. The hypothetical indicators measured in the three-dimensional (3D)/four-dimensional (4D) plots shown on the right side in the figure—a set taken from the large group of dimensionless indicators shown in the central column—are identified with descriptors that lack any known biological meaning: “*BAS*”, “*AB*”, and “*BBA*.” One example of a dimensionless indicator is the result from calculating: [M/L * N/M]/[N/L * L/M] over [M + L/N] * [L + N/M]/[N + M]/L * [M/N]. DPI: day(s) postinoculation with West Nile virus. Data source: Ref. (43).

**Figure 2:**
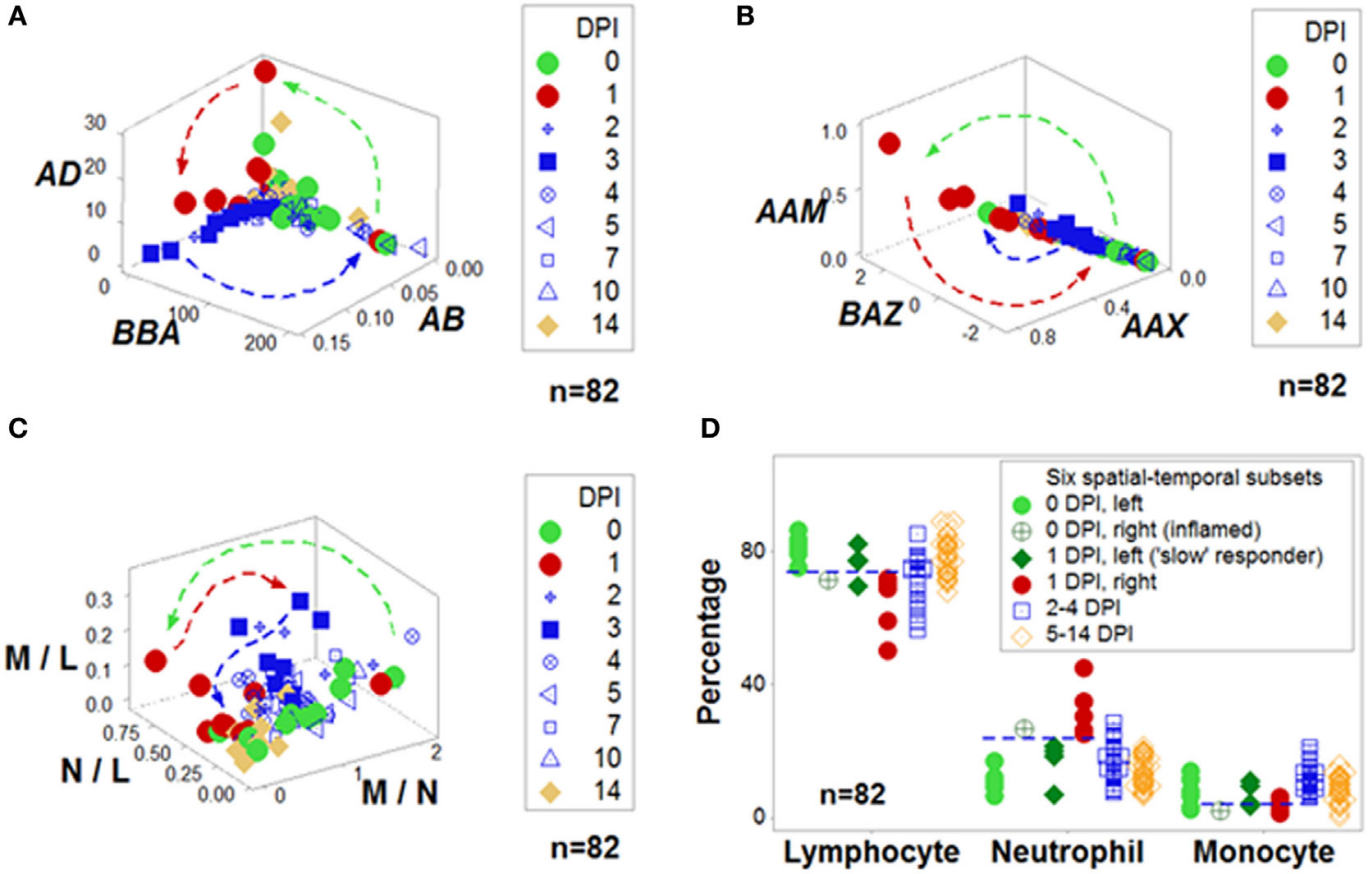
**Integration of non-reductionism and reductionism**. To both validate and interpret the non-reductionist graphic patterns (described in Figure 1), additional non-reductionist data analyses and reductionist (cell type-based) operations may be required. Highly complex data structures can demonstrate both discrimination and robustness **(A,B)**. In contrast, data structures of lower complexity may fail to distinguish changes that occur within 2 weeks **(C)**. Based on spatial–temporal patterns, numerous data subsets may be identified and interpreted. For instance, in this example, before challenge [0 day(s) postinoculation (DPI)], all birds but one were located on the left side of the plots displayed in Figure 1 [light green circles **(D)**]. In contrast, 24 h later (at 1 DPI), most challenged birds were on the right side [red symbols **(D)**]. However, some birds appeared to be “slow” responders: even at 1 DPI, they exhibited the profile of 0 DPI birds [dark green diamonds **(D)**]. The opposite profile was displayed by one 0 DPI animal, which revealed high neutrophil and low lymphocyte percentages [e.g., a profile indicative of an inflammation not due to the experimental challenge, dark, green circle with inserted cross **(D)**]. Inferences are facilitated by arrows that denote temporal data directionality **(A–C)** as well as non-overlapping data distributions [indicated by the horizontal lines **(D)**]. Because most data combinations have identical contents—except the three “words” [L and M, N and M, and L and N, shown in **(C)**], any other combination includes all data points of all three cell types **(A,B)**, information does not depend on data inputs (identical for all but three indicators) but relationships, e.g., three-dimensional/four-dimensional (spatial–temporal) data “shapes”, which can be rapidly validated and analyzed—as shown in the Movie S1 in Supplementary Material. Data source: Ref. (43).

Figure 1 outlines both reductionist and non-reductionist paradigms. It shows how combinations of few elements (cell types) can create numerous structures. Discrimination depends on pattern recognition—which, in turn, depends on complexity, i.e., the more *spatial–temporal* relationships captured, the higher the chances of differentiating data subsets. These concepts are explained with a mundane example: written language. While any “letter”, alone, lacks information (the left side of Figure 1), *combinations* of increasing complexity (“words”, “sentences”, “paragraphs”, “books”) possess meaning. When distinct *spatial* patterns emerge—such as the two perpendicular subsets exhibited by the upper 3D plot of Figure 1—and *temporal* data are considered (the lower plot shown on the right side of Figure 1, which displays 4D information), inferences can be based on arrows (*spatial*–*temporal* data *directionality*). While some inferences are visually obvious (Figures 2A,B), not all 4D patterns are interpretable, e.g., the use of the three basic “words” (M–L, N–L, and M–N interactions, expressed as ratios) cannot distinguish dynamics that take place within 2 weeks (Figure 2C). Yet, the spatial–temporal patterns shown in Figures 1 and 2A,B support the detection of, at least, six immunological data subsets (Figure 2D).

When emergent patterns are observed (which are not detected when reductionist approaches are utilized), one plausible inference is that they express *immunological functions* not previously recognized. As described in the Movie S1 in Supplementary Material, that hypothesis can be rapidly assessed.

Thus, non-reductionist data structures help discover preexisting functions (*propositional* knowledge). To validate such propositions, new tools or methods (*prescriptive* knowledge) may be required to conduct operations previously unfeasible (27, 62, 63).

## Non-Reductionist Applications

The postulates described in Figure 1 have been abundantly demonstrated (41, 44, 51, 52, 64). As shown in Figure 2C, data *ambiguity* may occur when structures of low complexity are used (41).

In contrast, new information emerges when highly complex data structures are utilized (Figures 2A,B; Movie S1 in Supplementary Material). The discriminant process follows the geometric criteria described by Gestalt psychologists 80 years ago, including *similarity, proximity, continuity, closure, common fate, parallelism*, and *symmetry* (61).

Non-reductionist, combinatorial approaches can both detect false-negative and -positive errors and differentiate early from late immune stages (51). They also distinguish subsets of septic patients that differ in mortality rates and immunological profiles (44).

Furthermore, non-reductionism can inform on patients empirically treated with antibiotics (41, 44). While reductionist tests do not evaluate *antimicrobial potency* and only provide *in vitro* (*antimicrobial susceptibility test-based*) data (4), non-reductionist methods can provide *earlier* (within 24 h) and *in vivo* information on antibiotic–immuno–microbial–temporal interactions (41). Because they may capture emergent (system-level) properties, non-reductionist analyses can yield more reliable results than those based on any one single factor (12).

Non-reductionist approaches can reveal interactions that involve cellular, supra-, and/or subcellular levels. Such approaches can simultaneously assess numerous *functions*, including (i) leukocyte activation, (ii) diapedesis, (iii) phagocytosis, (iv) early inflammation, and (v) the resolution phase of inflammation (64).

## The Future: Integration of Non-Reductionist and Reductionist Operations

The *one-to-many/many-to-one* “organizing principle” is ubiquitous: all vertebrates are protected from thousands of microbes by up to five leukocyte types. Even if all cell types—estimated to be approximately 210 (18)—performed antimicrobial functions, they could not fend off tens of thousands of microbes should only “one-to-one” (immunomicrobial) relationships exist. Clearly, the reductionist “single structure/single sequence/single function” theory is implausible (65).

In contrast, *multilevel functionality* seems to be one of Biology’s “first principles” (23, 66). Because it increases the complexity of the data—and, therefore, extracts more information—*multilevel functionality* may be operationalized by one-to-many/many-to-one constructs (32, 67).

However, non-reductionist approaches may generate artifacts. To validate such methods, reductionist *operations*—e.g., statistical analyses that focus on individual cell types—may be required (41, 44, 51, 52, 64).

Given the problems associated with reductionist concepts, the previous statement seems contradictory. Yet, it is not: a non-reductionist paradigm (an abstract entity) may be partially implemented by operations (concrete entities) that include reductionist procedures.

Integrated (non-reductionist and reductionist) constructs may improve experimental designs (68). Because experimental reductionism is inherently closed (69), it usually misses valuable information. In contrast, experiments conducted as a double (non-reductionist and reductionist) series of studies could circumvent the limitations of experimental reductionism.

## Conclusion

Because some properties of infectious disease-related data may possess undesirable consequences (e.g., data ambiguity prevents discrimination) and, in personalized medicine, decisions should be made even when the number of subjects *n* = 1, to diagnose and treat infectious diseases what is needed is not more data points (impossible when *n* = 1) but *temporal* data of greater *complexity*. To that end, immune profiles may be considered.

Because their repeatability can be easily determined—just a couple of studies can elucidate whether an observed immune pattern has been conserved across populations or species—immune profile-based inferences can measure *emergence*, i.e., patterns not shown by simple data structures that do not measure interactions—such as neutrophil percentages or counts—which may be revealed by 4D configurations of greater complexity (41).

A two-step procedure may detect and validate “emergence.” The first step is a non-reductionist, “top-down”, hypothesis-free, combinatorial process that creates numerous and complex indicators with the purpose of generating distinct (non-randomly distributed) data subsets (Figure 1). The second step is a reductionist (cell type-based) description of subsets meant to reveal, partially or totally, *non-overlapping* leukocyte data distributions which may also *differ temporally*. This double (spatial and temporal) data partitioning process is likely to be both *immunologically interpretable* and *statistically analyzable* (Figure 2D).

Integrated (non-reductionist and reductionist) approaches may fill the gap of the Oslerian bio-medical paradigm—which looks for correlations but does not investigate pathogenesis—and merge disciplines and technologies (30, 70, 71). Complex and dynamic (combinatorial) methods may be more predictable than classic (reductionist or linear) models (72, 73).

Hence, the major message of this review refers to the *generation and interpretation of biological information*. Because most combinations of immunological data include exactly the same contents (Figures 1 and 2; Movie S1 in Supplementary Material), *information does not depend on data inputs* but procedures that include non-reductionist and reductionist steps: (i) detection of distinct patterns, followed by (ii) biological validation and statistical analysis of the data subsets identified in the first step.

## Author Contributions

Conceived the study: AR. Contributed reagents/materials/data: GL, MJ, MI, SC, AI, SB, RP, and JCF. Wrote the paper: AR, AH, AA, JF, YA, and MR.

## Disclaimer

This study does not reflect the official positions and policies of the US EPA. Mention of products/trade names does not constitute recommendation for use by US EPA.

## Conflict of Interest Statement

While none of the authors received, at any time, any payment or services from a third party for any aspect of the submitted work, they wish to declare that they used a proprietary algorithm subject to a pending patent.

## Supplementary Material

Movie S1 **Biological reductionism vs. biological complexity**.
